# Association of Gastric Antrum Echodensity and Acute Gastrointestinal Injury in Critically Ill Patients

**DOI:** 10.3390/nu14030566

**Published:** 2022-01-27

**Authors:** Luping Wang, Hao Yang, Guangxuan Lv, Xin Fu, Yisong Cheng, Xi Zhong, Jing Yang, Bo Wang, Zhongwei Zhang, Xiaodong Jin, Yan Kang, Qin Wu

**Affiliations:** Department of Critical Care Medicine, West China Hospital, Sichuan University, No. 37 Guo Xue Road, Chengdu 610041, China; eeeeewlp@163.com (L.W.); nonamedoctor@163.com (H.Y.); 18846054403@163.com (G.L.); fffuxin1999@163.com (X.F.); chengyisong2020@wchscu.cn (Y.C.); zhongxivip2006@163.com (X.Z.); yjingscu@163.com (J.Y.); wchicu@126.com (B.W.); zhangzhongwei@scu.edu.cn (Z.Z.); zh_jxd@163.com (X.J.); kangyan@scu.edu.cn (Y.K.)

**Keywords:** gastric antrum echodensity, acute gastrointestinal injury, point-of-care ultrasound, feeding intolerance

## Abstract

(1) Background: Acute muscle inflammation leads to increased sonographic echodensity. We developed a technique to characterize the echodensity of the gastric antrum wall and assess its feasibility in evaluating the severity of acute gastrointestinal injury (AGI); (2) Methods: The B-mode images of the gastric antrum of each enrolled patient were obtained daily by point-of-care ultrasound (POCUS). The 50th percentile, 85th percentile, and mean value of the grayscale distribution according to histogram analysis (ED_50_, ED_85_, and ED_mean_, respectively) were used to characterize the gastric antrum echodensity. Consistency and correlation analyses were performed to evaluate the feasibility and reproducibility of gastric antrum echodensity measurement. The association of gastric antrum echodensity with the severity of AGI and its ability to predict feeding intolerance (FI) were analyzed; (3) Results: In total, 206 POCUS images of 43 patients were analyzed. The gastric antrum echodensity measurements had sufficient intra- and inter-investigator reliabilities (intraclass correlation coefficient >0.9 for all parameters). The ED_50_ showed a significant upward trend as AGI severity increased, as well as ED_85_ and ED_mean_ (*p* for trend <0.001, respectively). Patients who experienced FI had a higher ED_50_ (67.8 vs. 56.1, *p* = 0.02), ED_85_ (85.6 vs. 71.2, *p* = 0.01), and ED_mean_ (70.3 vs. 57.6, *p* = 0.01) upon enteral feeding initiation; (4) Conclusions: Measurement of gastric antrum echodensity was technically feasible and reproducible in ventilated patients. Increased gastric antrum echodensity was associated with greater severity of AGI. Patients with higher gastric antrum echodensity upon enteral nutrition initiation via a nasogastric tube were more likely to develop FI.

## 1. Introduction

Gastrointestinal (GI) dysfunction is a well-defined independent risk factor for mortality in critically ill patients [1]. Referring broadly to functional impairments of the GI tract such as poor motility and/or absorption, GI dysfunction may aggravate multiorgan failure and further deteriorate to life-threatening emergencies [2,3]. Efforts have been made to understand the underlying pathophysiology and perform daily monitoring of GI function in the intensive care unit (ICU) setting. As proposed by the European Society of Intensive Care Medicine Working Group on Abdominal Problems, the acute GI injury (AGI) grade has been widely used to define GI dysfunction in critically ill patients [4]. This concept is still valid, although it is generally not applicable in clinical studies because of its high observer dependency [1,5,6]. A new five-grade GI dysfunction score was developed to improve the predictive power for mortality based on the rationale of the previously developed AGI grade, but the score remained observer-dependent [7]. Biomarkers such as citrulline and intestinal fatty acid-binding protein have been unable to replace observer-dependent clinical assessment because of the precision of laboratory techniques and the threshold values chosen [8]. In summary, available monitoring techniques are relatively limited and management options are scarce [9].

Recent studies have demonstrated the potential for point-of-care ultrasound (POCUS) to provide a measure of GI function. POCUS enables assessment of GI tract features (GI diameter, GI wall thickness), contractility (gastric emptying, bowel peristalsis), and quality (echodensity) [10]. GI endpoints accessed by POCUS, including GI diameter, mucosal thickness, peristalsis, and blood flow, have been well defined and used to evaluate AGI and predict feeding intolerance (FI) by several studies [11,12]. However, both the GI and urinary tract sonography protocol and the simplified AGI ultrasonography score were relatively complicated, leading to an increased burden on the need for adequate training in order to achieve competency.

Tissue echodensity, also known as echogenicity, refers to the ability to reflect or transmit ultrasound waves in the context of surrounding tissues. Acute increases in muscle sonographic echodensity have been suggested to reflect muscle injury at a cellular level [13,14,15]. In the ICU setting, increases in diaphragm echodensity during the early course of mechanical ventilation have been associated with prolonged mechanical ventilation [16]. However, whether gastric antrum echodensity is associated with the severity of AGI in critically ill patients undergoing mechanical ventilation is unclear. This study was performed to characterize the gastric antrum echodensity, evaluate its relationship with the severity of AGI, and assess its ability to predict FI.

## 2. Materials and Methods

### 2.1. Study Design

This prospective study was conducted in the central ICU of West China Hospital located in Chengdu, Sichuan Province, China, and was approved by the West China Hospital of Sichuan University Biomedical Research Ethics Committee (No. 2021S673).

Patients who were admitted from July to August 2021 under mechanical ventilation were screened for eligibility. We excluded patients who were expected to die within 24 h after ICU admission and those from whom we could not obtain clear POCUS images for measurement of gastric antrum echodensity. Patients aged <18 years were also excluded. Written informed consent was obtained from all enrolled patients or their next-of-kin.

### 2.2. POCUS Examination and Echodensity Measurement

An experienced physician performed bedside POCUS to measure the gastric antrum and obtain B-mode images every morning before feeding for the first 7 days after ICU admission. All echodensity measurements of B-mode images were performed in triplicate, and the average scores were used in the final analyses. The gastric antrum was visualized using an ultrasound device with a 1- to 5-MHz curvilinear probe (CX50; Philips, Bothell, WA, USA). When obtaining images, time gain compensation was adjusted to the maximum, lateral gain compensation to zero, ultrasonic gain to 16, depth to 13 cm, and frequency to 38 Hz in accordance with the preset parameters for general abdominal examination. With the patient lying in the supine position, the gastric antrum was imaged on the parasagittal plane of the epigastric area using the left lobe of the liver, abdominal aorta, and superior mesenteric vein as internal markers [17]. POCUS B-mode images of the gastric antrum during terminal contraction were obtained for echodensity measurement. The anteroposterior diameter and craniocaudal diameter of the gastric antrum were measured between contractions, and the area of the gastric antrum was calculated as follows: cross-sectional area = π × anteroposterior diameter × craniocaudal diameter ÷ 4. Only ultrasound images that clearly showed the structure of the gastric antrum were considered to qualify for further analysis. During imaging processing, the color mode was converted to eight bit and the largest artifact-free area of the muscular layer of the gastric antrum was selected using ImageJ software 1.41o (National Institutes of Health, Bethesda, MD, USA). Histogram analysis of the echodensity of the selected region was performed, and the grayscale frequency distribution in the region was generated by ImageJ software. The grayscale was expressed as values between 0 (black) and 255 (white). The ED_50_, ED_85_, and ED_mean_ were defined as the 50th percentile, 85th percentile, and mean value of the grayscale distribution according to histogram analysis, respectively. Thirty images were randomly selected for a second measurement by the same investigator to assess the reproducibility of grayscale measurement. These images were also assessed by another independent investigator for the repeatability of grayscale measurement.

### 2.3. Data Collection

The following basic characteristics of all patients were collected at ICU admission: age, sex, weight, height, body mass index (BMI), diagnoses on ICU admission, comorbidities, Sequential Organ Failure Assessment (SOFA) score, and Acute Physiology and Chronic Health Evaluation II (APACHE II) score. Laboratory examination results were also collected, including the white blood cell count and the concentrations of hemoglobin, albumin, total bilirubin, serum creatinine, glucose, C-reactive protein, procalcitonin, and lactic acid. Each patient’s GI function was evaluated daily and assigned one of four grades of AGI severity for the first 7 days after admission, also assessed by using Gastrointestinal Dysfunction Score (GIDS) and Gastrointestinal Failure (GIF) score system [4,7,18]. Information regarding ICU interventions was collected including the cumulative dose of sedation/analgesia, including midazolam, propofol, dexmedetomidine, remifentanil, and sufentanyl within the first week and prokinetics use, norepinephrine use, and vasopressin use during follow-up, 28-day ventilation-free days (number of days of successful liberation from ventilation within 28 days after ICU admission), and 28-day mortality rate.

### 2.4. Nutrition Delivery

Enteral nutrition (EN) was delivered at 20 mL/h on the first day. The feeding rate was increased for patients who tolerated feeding well, but it did not exceed 150 mL/h. On the next day and subsequent days, patients were evaluated daily to determine whether transition to a volume-based feeding strategy was suitable based on the Enhanced Protein-Energy Provision via the Enteral Route in Critically Ill Patients protocol [19]. The protein target was 1.2 to 2.0 g/kg/day. If EN did not meet 60% of the energy and protein targets after 7 days, additional parenteral nutrition supplementation was considered.

FI was defined as the occurrence of the following symptoms resulting in forced interruption of EN: vomiting or regurgitation, diarrhea, ileus, and mesenteric ischemia/perforation. Vomiting/regurgitation was diagnosed if any visible reflux of gastric contents occurred. Diarrhea was defined as loose or liquid stools three or more times a day, with a stool weight of >200 to 250 g/d and difficulty in correcting the condition by administering medications and managing EN formulas. Ileus was diagnosed in patients with a cecum diameter of >9 cm or a colon diameter of >6 cm. Diagnosis of mesenteric ischemia/perforation was based on positive signs on abdominal computed tomography [20]. During follow-up, FI was assessed daily by two independent attending physicians with at least 5 years of medical experience.

### 2.5. Statistical Analysis

Continuous variables are presented as mean ± standard deviation or median (interquartile range), and categorical variables are presented as number (percentage). Differences in continuous variables were compared using Student’s t-test or the Mann–Whitney U test. Reproducibility and repeatability of echodensity measurement were tested by performing the Bland–Altman plot and calculating the intraclass correlation coefficient (ICC). Spearman’s rank correlation was conducted to analyze correlations between each gastric antrum echodensity biomarker (ED_50_, ED_85_, and ED_mean_) and the four grades of AGI severity. The median ED_50_, ED_85_, and ED_mean_ of each AGI grade were input separately into the linear logistic regression model as continuous variables to determine whether a linear trend was present between the four grades of AGI severity and each echodensity biomarker. Multivariate logistic regression analysis was performed to adjust for several confounders. In each model, the median odds ratio (OR) of each AGI grade was input separately into the linear regression model as a continuous variable to determine whether a linear trend was present between the risk of an increase in each echodensity biomarker (ED_50_, ED_85_, and ED_mean_) and the four grades of AGI severity. A receiver operating characteristic curve was performed to calculate the threshold of each echodensity biomarker, and the Youden index was used to determine the ability to predict FI. The area under the receiver operating characteristic curve (AUC) was applied to assess the predictive ability of each echodensity biomarker, with FI as the result of interest. A two-tailed *p*-value of <0.05 was considered statistically significant. All statistical analyses were performed using SPSS 26.0 (IBM Corp., Armonk, NY, USA) and R version 4.0.3 (https://www.r-project.org/. accessed on 14 November 2021).

## 3. Results

### 3.1. Participants

**Figure 1:**
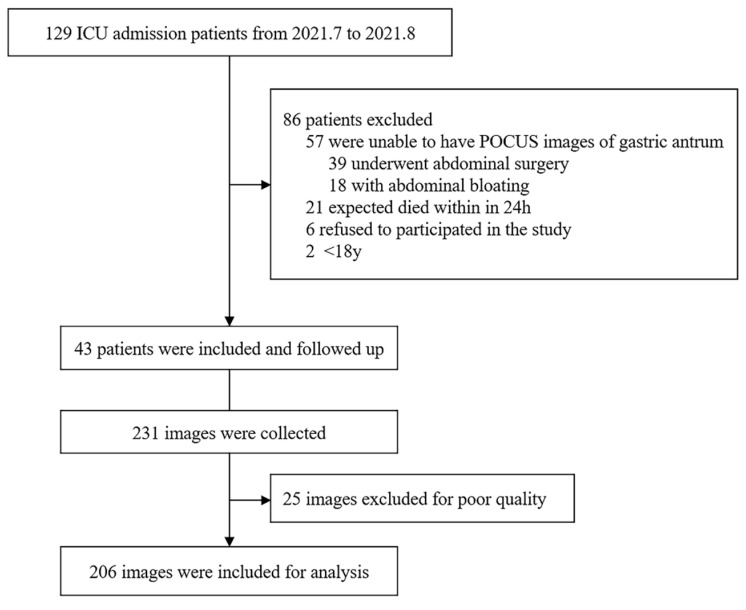
Flowchart and enrollment of patients. ICU: intensive care unit; POCUS: point-of-care ultrasound.

In total, 129 patients were screened from July to August 2021. We excluded 57 patients who were unable to undergo POCUS imaging of the gastric antrum (39 patients who underwent abdominal surgery and 18 patients with abdominal bloating), 21 patients who were expected to die within 24 h after ICU admission, 6 patients who refused to participate in this study, and 2 patients aged <18 years at admission. The remaining 43 patients were finally enrolled in this study (Figure 1). The patients’ mean age was 60.1 ± 14.2 years, and they comprised 28 (65.1%) men and 15 (34.9%) women. The mean APACHE II score and SOFA score were 18.4 ± 6.2 and 9.4 ± 3.8, respectively. The overall mortality rate was 23.3% (n = 10). The characteristics of the study population are shown in Table 1. In this cohort, 13 AGI grade 0 events, 23 grade I events, 46 grade II events, 70 grade III events, and 54 grade IV events were observed.

### 3.2. Measurement of Gastric Antrum Echodensity and Consistency Analysis

**Figure 2:**
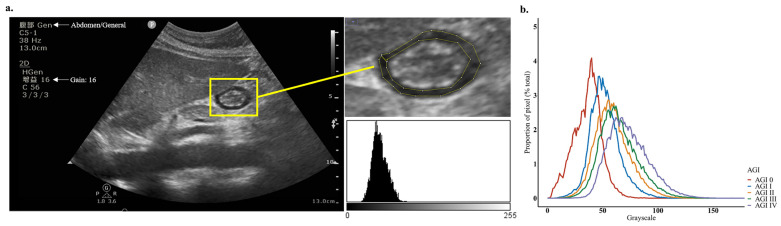
(**a**) The POCUS image of gastric antrum echodensity as processed by the ImageJ with histogram analysis; (**b**) grayscale distribution of varied AGI grades. The grayscale distribution represents the average proportion of pixels (percentage of the total pixels) at each grayscale intensity of gastric antrum echodensity images in varied severities of AGI. POCUS: point-of-care ultrasound, AGI: acute gastrointestinal injury.

In total, 231 POCUS images of the gastric antrum were available for all patients, and 24 images were excluded because of poor quality. Therefore, 206 POCUS images were analyzed (Figure 1). The echodensity was measured using our protocol (Figure 2a). The measurement of anteroposterior diameter and craniocaudal diameter is shown in Figure S1. The Bland–Altman plots showed that the mean difference of ED_50_ was 0.33 (95% confidence interval [CI]: −1.99–2.65), ED_85_ was 0.8 (95% CI: −2.51–4.11), and ED_mean_ was 0.5 (95% CI: −1.52–2.52) as measured twice by the same investigator. The Bland–Altman plots showed that the mean difference of ED_50_ was −0.37 (95% CI: −3.3–2.57), ED_85_ was −0.57 (95% CI: −6.61–5.47), and ED_mean_ was −0.32 (95% CI: −3.9–3.27) as measured twice by independent investigators (Figure S2). The ICC (95% CI) for reproducibility was 0.992 (0.982–0.996) for ED_50_ measurement (*p* < 0.001), 0.986 (0.965–0.994) for ED_85_ measurement (*p* < 0.001), and 0.993 (0.983–0.997) for ED_mean_ measurement (*p* < 0.001). The ICC (95% CI) for repeatability was 0.987 (0.973–0.994) for ED_50_ measurement (*p* < 0.001), 0.962 (0.923–0.982) for ED_85_ measurement (*p* < 0.001), and 0.983 (0.964–0.992) for ED_mean_ measurement (*p* < 0.001).

### 3.3. Association between Gastric Antrum Echodensity and AGI Grade

**Figure 3:**
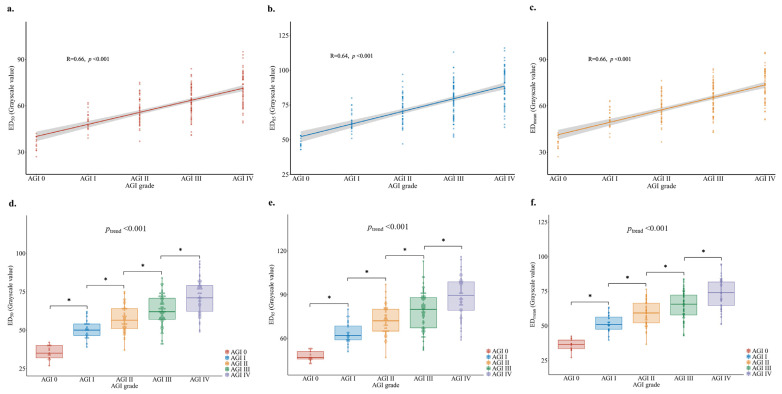
(**a**–**c**). Correlation of ED_50_, ED_85_, ED_mean_ with AGI grade; (**d**–**f**). Boxplot of the change of ED_50_, ED_85_, ED_mean_ with AGI grade. The trend test is mainly based on the median ED_50_, ED_85_, and ED_mean_ of each AGI grade, which were input separately into the linear logistic regression model as continuous variables. AGI: acute gastrointestinal injury. *: *p*-value < 0.05.

The gray distribution represents the average percentage of pixels in each AGI grade (Figure 2b). The medians of the gray distribution curve of AGI grade 0, I, II, III, and IV events were 37, 50, 58, 62, and 71, respectively. An increased ED_50_, ED_85_, and ED_mean_ was significant correlated with AGI severity in all critically ill patients studied (*p* < 0.001 respectively, Figure 3a–c). The ED_50_ showed a significant upward trend as AGI severity increased (median: 35 for AGI 0, 50 for AGI I, 56.5 for AGI II, 62 for AGI III, and 71 for AGI IV, *p* for trend < 0.001, Figure 3d). The ED_85_ also showed a significant upward trend as AGI severity increased (median: 47 for AGI 0, 62 for AGI I, 72 for AGI II, 80 for AGI III, and 89.5 for AGI IV, *p* for trend < 0.001, Figure 3e), as well as the ED_mean_ (median: 36.6 for AGI 0, 51 for AGI I, 59.3 for AGI II, 65.6 for AGI III, and 73.9 for AGI IV, *p* for trend <0.001, Figure 3f). Additionally, there were significant associations between the AGI grade and the studied ED variables on different days (Figure S3). Significant upward trends were also seen in GIDS with ED_50_, ED_85_ and ED_mean_, as well as in GIF with ED_50_, ED_85_ and ED_mean_ (*p* for trend <0.001 respectively, Figure S4). Multivariate logistic regression analysis showed that an increasing ED_50_ (OR = 2.3, 95% CI: 1.5–3.6), ED_85_ (OR = 2, 95% CI: 1.4–2.7), and ED_mean_ (OR = 2.2, 95% CI: 1.5–3.3) were each associated with an increasing risk of higher severity of AGI (*p* for trend < 0.001, Table 2). This positive correlation of ED_50_, ED_85_, and ED_mean_ with the severity of AGI persisted when we adjusted for age (*p* for trend <0.001), for age and BMI (*p* for trend < 0.001), and for age, BMI, APACHE II score, and SOFA score at ICU admission (*p* for trend < 0.001).

### 3.4. Ability to Predict FI

Thirty-eight patients started enteral feeding during the first 7 days of their ICU stay, and 16 patients developed FI (Table S1). The baseline characteristics of patients in the Non-FI and FI groups are shown in Table 3. The mean ED_50_ on the first day of EN delivery was 56.1 ± 12.7 in the non-FI group and 67.8 ± 11.1 in the FI group (*p* = 0.02). The mean ED_85_ on the first day of EN delivery was 71.2 ± 13.5 in the non-FI group and 85.6 ± 14.9 in the FI group (*p* = 0.01). The mean ED_mean_ was 57.6 ± 12.9 in the non-FI group and 70.3 ± 11.3 in the FI group (*p* = 0.01). The threshold of ED_50_ to predict FI was 63 (specificity: 87.5%, sensitivity: 69.2%) with an AUC of 0.76 (95% CI: 0.57–0.94, *p* = 0.006) at EN initiation. The threshold of ED_85_ to predict FI was 77.5 (specificity: 75.0%, sensitivity: 69.2%) with an AUC of 0.75 (95% CI: 0.57–0.93, *p* = 0.006). The threshold of ED_mean_ to predict FI was 65.9 (specificity: 87.5%, sensitivity: 69.2%) with an AUC of 0.76 (95% CI: 0.59–0.94, *p* = 0.004) (Figure S5).

## 4. Discussion

In this study, we developed a technique for measuring gastric antrum echodensity and explored the association of gastric antrum echodensity with the AGI grade in critically ill patients. We found that the measurement of gastric antrum echodensity is feasible with acceptable reproducibility and repeatability in patients undergoing mechanical ventilation. In the present study, increased gastric antrum echodensity was associated with greater AGI severity. Patients with higher gastric antrum echodensity upon EN initiation via a nasogastric tube were more likely to develop FI.

Gastric motility disorders play a key role in GI dysfunction in critically ill patients and generally manifest as gastric antrum motility disorders, delayed gastric emptying, and diminished migrating motor complexes [21,22]. The gastric residual volume (GRV) has been widely used to monitor delayed gastric emptying. However, current studies suggest that monitoring of the GRV does not reduce the risk of adverse events but rather reduces the delivery of EN in critically ill patients [23,24]. Quantitative assessment of gastric function remains limited. In this study, we developed a novel technique to qualify gastric antrum echodensity in critically ill patients for the purpose of characterizing gastric function. Normal muscle tissue appears black with low echodensity [25]. Increased muscle echodensity has been reported in loss of healthy muscle structure (replacement of aging or diseased muscle tissue by fat and fibrous tissue) and inflammatory cell infiltration [14,25,26,27]. Several studies have indicated that increased muscle echodensity reflects early muscle injury in critical illness [13,28,29]. Other studies have shown that increased diaphragm echodensity is associated with a prolonged duration of mechanical ventilation and that increased echodensity in the diaphragm and rectus femoris are negatively correlated with mortality in critically ill patients [16,30,31]. Taken together, these findings suggest that increased muscle echodensity is correlated with declining quality and dysfunction of muscle, confirming the potential of gastric antrum echodensity to characterize gastric function.

Our results suggest that the measurement of gastric antrum echodensity is a highly feasible and reliable method to evaluate the severity of AGI within and between investigators. Echodensity measurement was performed in the maximum no-artifact zone during gastric antrum contraction to avoid the interference of artifacts with GI muscle density as much as possible. The grayscale distribution of the gastric antrum generally shows a normal distribution. However, it may change to a skewed distribution as a result of inhomogeneous changes in muscle structure during disease processes. Therefore, we identified and recorded parameters including both mean and percentile grayscale values (ED_50_, ED_85_, and ED_mean_) to describe echodensity changes in critically ill patients. Our results indicated that all three grayscale parameters were reliable to evaluate the severity of AGI.

We observed increased gastric antrum echodensity in patients with higher AGI grades. Similar trends were observed in GIDS or GIF score system. Considering the influence of age [26,32], BMI, and disease severity on echodensity measurement, we adjusted for these factors in the multivariate logistic regression models and found that the association between gastric antrum echodensity and AGI grade remained. Multiple factors such as intestinal nerve disorders, smooth muscle dysfunction, inflammatory infiltration, surgical factors, ICU intervention, and disease severity can induce direct or indirect damage to GI muscles in critically ill patients [21,22]. The specific pathophysiological process of increased echodensity in the gastric antrum remains unclear. One explanation is that gastric edema is induced by inflammation and capillary leakage as well as gastric smooth muscle fibrosis and fatty degeneration. Muscle edema in the acute stage and fibrosis and fatty degeneration in the subacute stage are reportedly correlated with increased muscle echodensity in patients with sepsis [29]. The severity of muscle edema and positive cumulative fluid balance may affect the interpretation of muscle echodensity [16,29,31]. Further studies involving tissue biopsies are needed to explore the mechanism of increased echodensity after occurrence of AGI.

In addition to the association between gastric antrum echodensity and AGI grade, we also found that the patients who developed FI had a significantly higher gastric antrum echodensity upon enteral feeding initiation. This result suggests that patients with higher gastric antrum echodensity have more difficult EN delivery and a higher risk of GI adverse events. In such patients, EN should be delivered with more caution. In our cohort, we observed that SOFA score and mortality in the non-FI group were higher than those in the FI group, which may be caused by the fact that several patients died within a short time after EN initiation because of their severity and deterioration of their condition and were allocated to the non-FI group because no FI events occurred in a short feeding time. Another possible reason is the small sample size. We also observed that there was no statistical difference in antral area between the two groups, whereas previous study suggested that increased antral area was associated with FI [33]. One reason for this might be that we measured the area of the gastric antrum before feeding, whereas the previous study measured it when feeding was ongoing or planned to continue. In addition, FI may be caused by different parts of the GI tract [34], so it may occur without an increase in gastric antrum area. At the same time, the small sample size may also have affected the accuracy of the results. The predictive ability of gastric antrum echodensity in predicting FI was moderate, and the 95% CI of the AUC of each grayscale parameter was wide because of the sample size. Gastric antrum echodensity might serve as a clinical predictor of the occurrence of FI at EN initiation and guide the management of FI. Gastric antrum echodensity combined with other GI endpoints determined by POCUS may provide more comprehensive and accurate information on anatomical and functional GI injury in critically ill patients. Further studies are needed to standardize the POCUS protocol and apply it to the evaluation of GI dysfunction.

This is the first study to describe a novel technical method to assess GI dysfunction in patients undergoing mechanical ventilation. However, the study has some limitations. This was a single-center prospective study with a small sample size, which may have influenced the accuracy of the results. The use of ultrasonic examination with different internal performance parameter settings and gain and depth parameters may have influenced the measurement of gastric antrum echodensity. We only explored the association between gastric antrum echodensity and AGI grade and the influence of intestine function was not included. The abdominal conditions may have interfered with image acquisition and observer dependency. Fifty-seven (44%) patients were excluded because POCUS images of the gastric antrum were not available, including 18 (14%) patients who could not have POCUS images because of abdominal bloating. Therefore, the measurement of gastric antrum echodensity cannot completely replace other methods for evaluating GI dysfunction. Although we adjusted for some confounding factors in the multivariable regression, other overlooked confounding factors may have affected the results. The histogram analysis of the current echodensity could not be obtained immediately at the bedside.

## 5. Conclusions

The technical measurement of gastric antrum echodensity was highly feasible and reproducible in evaluating the severity of AGI in critically ill patients undergoing mechanical ventilation. Increased gastric antrum echodensity was associated with higher AGI grades. Patients who developed FI also had significantly higher gastric antrum echodensity on the first day of enteral feeding. Gastric antrum echodensity might be able to predict the occurrence of FI at EN initiation and guide the management of FI in critically ill patients.

## Figures and Tables

**Table 1:** The basic information and characterization of enrollment patients.

Characteristic	Total (n = 43)
Age (years), mean ± SD	60.1 ± 14.2
Male Sex, No. (%)	28 (65.1)
BMI, mean ± SD	23.3 ± 3.4
APACHE II score, mean ± SD	18.4 ± 6.2
SOFA score, mean ± SD	9.4 ± 3.8
Diagnosis, No. (%)	
Sepsis	14 (32.6)
espiratory failure	10 (23.3)
Multiple trauma	6 (14)
Others ^1^	13 (30.2)
Comorbidities, No. (%)	
Cardiovascular Diseases ^2^	17 (39.5)
Neurological Diseases ^3^	15 (34.9)
Diabetes	6 (14)
Laboratory tests at admission, mean ± SD	
White blood cell (×10^9^/L)	11.1 ± 7.3
Hemoglobin (g/L)	91.2 ± 22
Albumin (g/L)	31.3 ± 5.9
Total bilirubin (umol/L)	31.6 ± 45.3
Serum creatinine (umol/L)	136 ± 191
Glucose (mmol/L)	9.2 ± 4.3
C-reactive protein (mg/L)	90 ± 68.5
Procalcitonin (μg/L)	5.3 ± 9.2
Lactic acid (mmol/L)	2.2 ± 1.8
ICU intervention	
Norepinephrine use (%)	25 (58.1)
Vasopressin use (%)	7 (16.3)
Prokinetics use (%)	15 (34.9)
Cumulative sedation and analgesia dose within first week, mean ± SD	
Midazolam (mg/kg)	5.9 ± 8.6
Propofol (mg/kg)	48.7 ± 60.2
Dexmedetomidine (μg/kg)	17.9 ± 22.7
Remifentanil (mg/kg)	0.3 ± 0.3
Sufentanyl (μg/kg)	4.1 ± 10
AGI score on ICU admission	
AGI 0	2 (4.7)
AGI I	9 (20.9)
AGI II	3 (7)
AGI III	17 (39.5)
AGI IV	12 (27.9)
GIDS on ICU admission	
GIDS 0	10 (23.3)
GIDS 1	25 (58.1)
GIDS 2	6 (14)
GIDS 3	2 (4.7)
GIF score on ICU admission	
GIF 0	7 (16.3)
GIF 1	34 (79.1)
GIF 2	2 (4.7)
28 d ventilation-free days (d)	20.1 ± 5.1
28 d mortality	10 (23.3)

SD: standard deviation, BMI: body mass index, APACHE II: Acute Physiology and Chronic Health Evaluation II, SOFA: Sequential Organ Failure Assessment, ICU: intensive care unit, AGI: acute gastrointestinal injury, GIDS: Gastrointestinal Dysfunction Score, GIF: Gastrointestinal Failure. ^1^ Others include epilepsy, heat stroke, acute myocardial infarction, mitral valve perforation, autoimmune hemolytic anemia, poisoning, alcoholism, multiform erythema, malignant tumor, end-stage renal disease. ^2^ Cardiovascular diseases include coronary heart disease, hypertension, infectious endocarditis, arrhythmia, cardiac failure, and pericardial effusion. ^3^ Neurological diseases include hypoxic-ischemic encephalopathy, epilepsy, cerebral contusion, subarachnoid hemorrhage, brain atrophy, cerebral infarction, and intracranial hematoma.

**Table 2:** Multivariable-adjusted association of ED_50_, ED_85_, ED_mean_ with the severity of AGI grade.

Variable	AGI Grade					*p* for Trend
	AGI 0	AGI I	AGI II	AGI III	AGI IV	
ED_50_						
Median grayscale (IQR)	35 (31.5–40)	50 (46–54)	56.5 (50.8–64)	62 (57–71)	71 (62–79)	
Unadjusted OR (95%CI)	1 (Ref)	1.8 (1.2–2.7)	2 (1.3–3.1)	2.1 (1.4–3.3)	2.3 (1.5–3.6)	<0.001
Model 1 OR (95%CI)	1 (Ref)	1.8 (1.1–2.9)	2 (1.3–3.2)	2.2 (1.4–3.5)	2.4 (1.5–3.8)	<0.001
Model 2 OR (95%CI)	1 (Ref)	1.9 (1.1–3.3)	2.1 (1.2–3.5)	2.3 (1.3–3.8)	2.5 (1.5–4.2)	<0.001
Model 3 OR (95%CI)	1 (Ref)	2.2 (1.1–4.5)	2.6 (1.3–5.3)	2.9 (1.4–6)	3.2 (1.6–6.6)	<0.001
ED_85_						
Median grayscale (IQR)	47 (45.5–52)	62 (59–69)	72 (64.8–80)	80 (67–88)	89.5 (79–99)	
Unadjusted OR (95%CI)	1 (Ref)	1.6 (1.2–2.2)	1.8 (1.3–2.5)	1.9 (1.3–2.6)	2 (1.4–2.7)	<0.001
Model 1 OR (95%CI)	1 (Ref)	1.7 (1.2–2.5)	1.9 (1.3–2.7)	2.0 (1.4–2.9)	2.1 (1.4–3.1)	<0.001
Model 2 OR (95%CI)	1 (Ref)	1.8 (1.2–2.7)	1.9 (1.3–2.9)	2.0 (1.3–3)	2.1 (1.4–3.2)	<0.001
Model 3 OR (95%CI)	1 (Ref)	2.8 (1–8)	3.2 (1.1–9.2)	3.5 (1.2–10)	3.7 (1.3–10.7)	<0.001
ED_mean_						
Median grayscale (IQR)	36.6 (33–40.3)	51 (47.2–57.1)	59.3 (52.1–66.6)	65.6 (57.8–72.4)	73.9 (64.5–81.9)	
Unadjusted OR (95%CI)	1 (Ref)	1.7 (1.2–2.5)	1.9 (1.3–2.8)	2 (1.4–3)	2.2 (1.5–3.3)	<0.001
Model 1 OR (95%CI)	1 (Ref)	1.8 (1.2–2.7)	2 (1.3–3)	2.1 (1.4–3.2)	2.3 (1.5–3.5)	<0.001
Model 2 OR (95%CI)	1 (Ref)	1.9 (1.2–3)	2 (1.3–3.3)	2.2 (1.3–3.5)	2.4 (1.5–3.8)	<0.001
Model 3 OR (95%CI)	1 (Ref)	2 (1.1–3.5)	2.3 (1.3–4.1)	2.6 (1.5–4.5)	2.8 (1.6–5)	<0.001

OR and 95% CI were calculated with the use of a multiple logistic regression model. Model 1 was adjusted for age. Model 2 was adjusted for age and BMI. Model 3 was adjusted for age, BMI, APACHE II score and SOFA score at ICU admission. The trend test is mainly based on the variable median input linear regression model. AGI: acute gastrointestinal injury, BMI: body mass index, IQR: interquartile range, OR: odds ratio, CI: confidence interval, APACHE II: Acute Physiology and Chronic Health Evaluation II, SOFA: Sequential Organ Failure Assessment.

**Table 3:** Baseline and characteristics of patients in Non-FI and FI group.

Characteristic	Total (n = 38)	Non-FI Group (n = 21)	FI Group (n = 17)	*p*-Value
Age (years), mean ± SD	61 ± 14.4	57.7 ± 13.9	65.1 ± 14.5	0.12
Male Sex, No. (%)	24 (63.2)	13 (61.9)	11 (64.7)	0.86
BMI, mean ± SD	23.2 ± 3.6	23.4 ± 4	22.9 ± 3.2	0.66
APACHE II score, mean ± SD	18.6 ± 6	19.2 ± 6.2	17.9 ± 5.8	0.5
SOFA score, mean ± SD	9.4 ± 3.6	10.4 ± 4.1	8.1 ± 2.4	0.051
Diagnosis, No. (%)				
Sepsis	11 (28.9)	5 (23.8)	6 (35.3)	0.68
Respiratory failure	10 (26.3)	6 (28.6)	4 (23.5)	>0.99
Multiple trauma	4 (10.5)	3 (14.3)	1 (5.9)	0.76
Others ^1^	13 (34.2)	7 (33.3)	6 (35.3)	0.9
Comorbidities—No. (%)				
Cardiovascular diseases ^2^	16 (42.1)	5 (23.8)	11 (64.7)	0.01
Neurological diseases ^3^	14 (36.8)	8 (38.1)	6 (35.3)	0.86
Diabetes	6 (15.8)	3 (14.3)	3 (17.6)	>0.99
Cumulative sedation and analgesia dose within first week, mean ± SD				
Midazolam (mg/kg)	6.6 ± 8.9	8.6 ± 10	4.1 ± 6.9	0.12
Propofol (mg/kg)	53.6 ± 62.1	63.6 ± 69.1	41.3 ± 51.5	0.28
Dexmedetomidine (μg/kg)	20.1 ± 23.3	16.2 ± 24.5	24.9 ± 21.6	0.26
Remifentanil (mg/kg)	0.3 ± 0.3	0.4 ± 0.4	0.2 ± 0.3	0.13
Sufentanyl (μg/kg)	4.3 ± 10.6	3.6 ± 11.1	5.3 ± 10.1	0.63
Time to EN initiation from ICU admission (h), mean ± SD	56.4 ± 54.7	52.1 ± 50.3	61.7 ± 60.9	0.59
Prokinetics use (%)	15 (39.5)	7 (33.3)	8 (47.1)	0.26
Norepinephrine (μg/kg/min) at EN initiation, mean ± SD	0.1 ± 0.2	0.11 ± 0.2	0.08 ± 0.16	0.65
Ultrasonic parameters at EN initiation, mean ± SD				
ED_50_	61.3 ± 13.2	56.1 ± 12.7	67.8 ± 11.1	0.02
D_85_	77.7 ± 15.7	71.2 ± 13.5	85.6 ± 14.9	0.01
ED_mean_	63.3 ± 13.6	57.6 ± 12.9	70.3 ± 11.3	0.01
Anteroposterior diameter (cm)	2.4 ± 0.6	2.4 ± 0.6	2.4 ± 0.6	0.98
Craniocaudal diameter (cm)	3 ± 0.7	3 ± 0.5	3 ± 0.9	0.87
Antral area (cm^2^)	5.6 ± 2.1	5.6 ± 1.7	5.7 ± 2.7	0.87
28 d ventilation-free days (d), mean ± SD	20.1 ± 5.1	21.8 ± 4.4	19.1 ± 5.8	0.31
28 d mortality (%)	8 (21.1)	6 (28.6)	2 (11.8)	0.39

All *p* values are 2-tailed. SD: standard deviation, BMI: body mass index, APACHE II: Acute Physiology and Chronic Health Evaluation II, SOFA: Sequential Organ Failure Assessment, EN: enteral nutrition, ICU: intensive care unit. ^1^ Others include including epilepsy, heat stroke, acute myocardial infarction, mitral valve perforation, autoimmune hemolytic anemia, poisoning, alcoholism, multiform erythema, malignant tumor, end-stage renal disease. ^2^ Cardiovascular diseases include coronary heart disease, hypertension, infectious endocarditis, arrhythmia, cardiac failure, and pericardial effusion. ^3^ Neurological diseases include hypoxic-ischemic encephalopathy, epilepsy, cerebral contusion, subarachnoid hemorrhage, brain atrophy, cerebral infarction, and intracranial hematoma.

## Data Availability

The data sets used and/or analyzed during the current study are available from the corresponding author on reasonable request.

## Supplementary Materials

The following supporting information can be downloaded at: https://www.mdpi.com/article/10.3390/nu14030566/s1, Figure S1: Measurements of anteroposterior diameter and craniocaudal diameter of the gastric antrum. (a) anteroposterior diameter, (b) craniocaudal diameter; Figure S2: Bland–Altman plots of consistency analysis. (a–c) Bland–Altman plots of ED_50_, ED_85_, ED_mean_ measured in the first and second tests by the same investigator. (d–f) Bland–Altman plots of ED_50_, ED_85_, ED_mean_ measured in the first and second investigator tests; Figure S3: (a–g) Box plots of ED_50_, ED_85_, and ED_mean_ for varied AGI grades in the first 7 days after ICU admission. The trend test is mainly based on the median ED_50_, ED_85_, and ED_mean_ of each AGI grade on different days, which were input separately into the linear logistic regression model as continuous variables. AGI: acute gastrointestinal injury, ICU: Intensive care unit. *: *p*-value < 0.05; Figure S4: Boxplot of the change of ED_50_, ED_85_, ED_mean_ with GIDS and GIF score. The trend test is mainly based on the median ED_50_, ED_85_, and ED_mean_ of each GIDS grade (a–c) or GIF grade (d–f), which were input separately into the linear logistic regression model as continuous variables. GIDS score: Gastrointestinal Dysfunction Score; GIF score: Gastrointestinal Failure score; Table S1: Detailed description of feeding intolerance. FI: feeding intolerance; Figure S5: The receiver operating characteristic curve of ED_50_, ED_85_ and ED_mean_ in predicting feeding intolerance at enteral nutrition initiation.
