# Supplementary material for: Association of Gastric Antrum Echodensity and Acute Gastrointestinal Injury in Critically Ill Patients
Source: Nutrients. 2022 Jan 27;14(3):566. doi: 10.3390/nu14030566 (PMC8838069; doi:10.3390/nu14030566)
Supplement: Supplementary file 1 [file nutrients-14-00566-s001.zip › nutrients-1565952-supplementary.pdf]

nutrients

Supplementary Materials

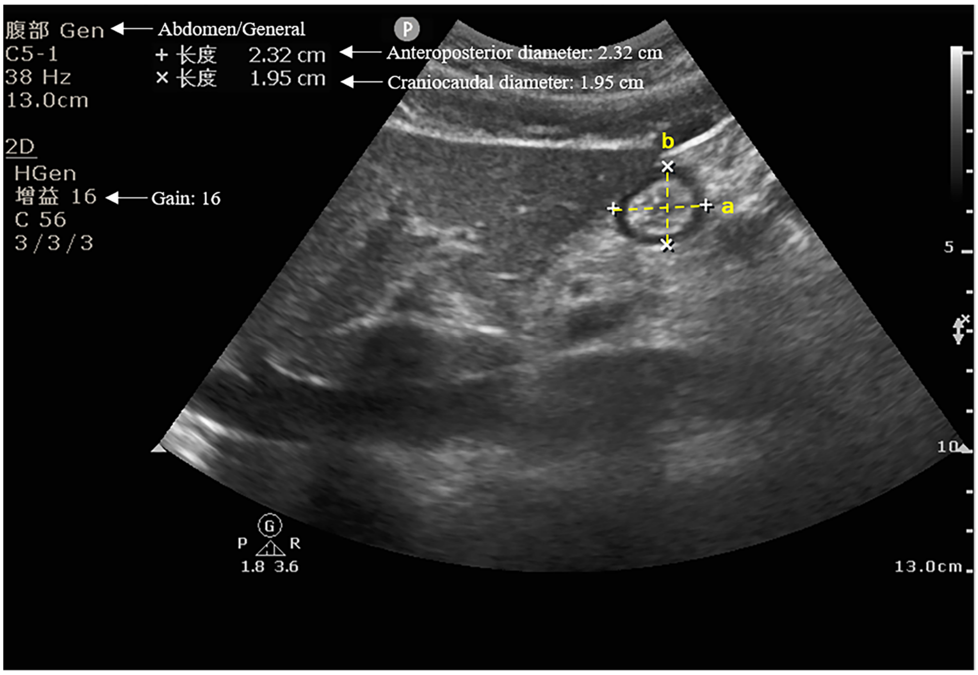

Figure S1. Measurements of anteroposterior diameter and craniocaudal diameter of the gastric antrum. **a.** anteroposterior diameter, **b.** craniocaudal diameter.

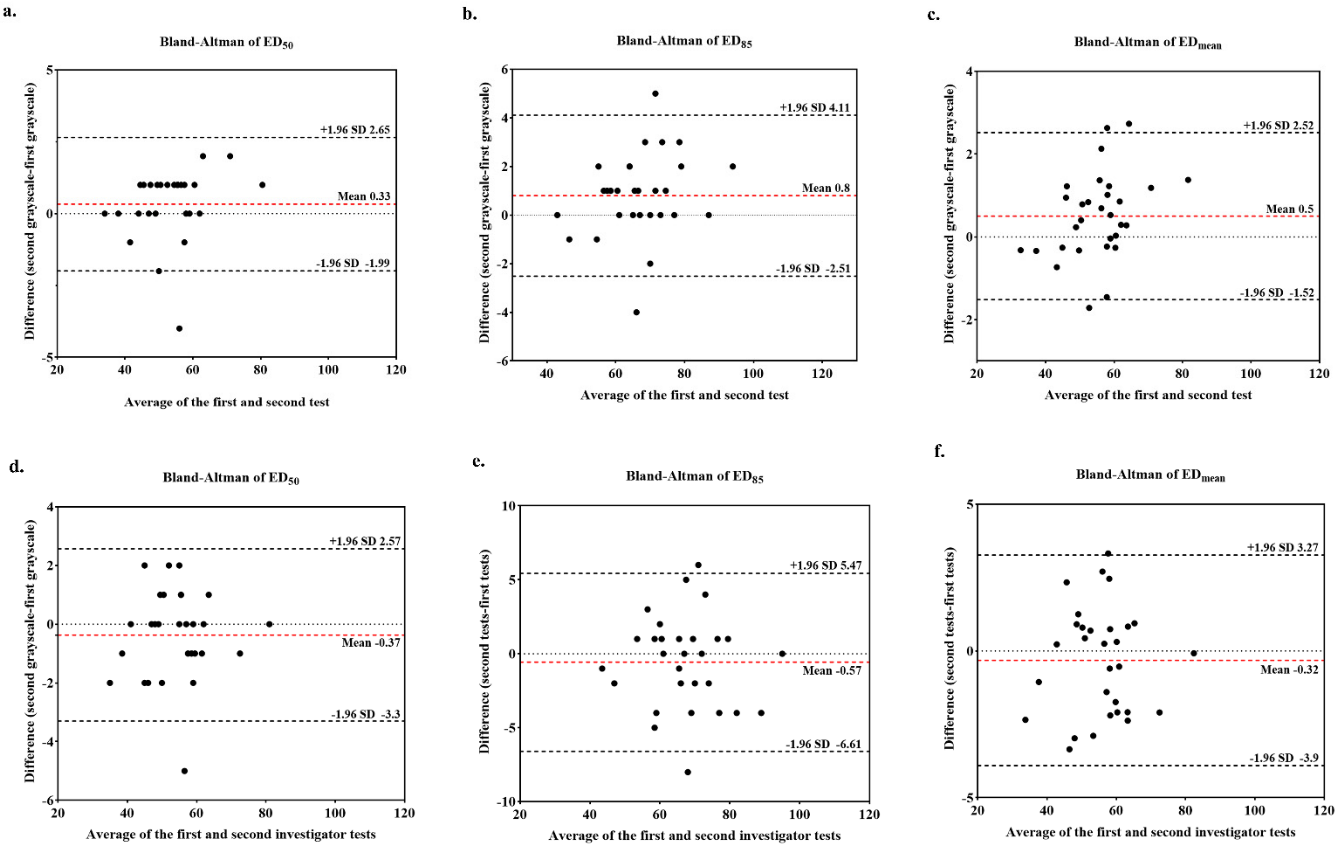

Figure S2. Bland–Altman plots of consistency analysis. (a–c) Bland–Altman plots of ED_{50}, ED_{85}, ED_{mean} measured in the first and second tests by the same investigator. (d–f) Bland–Altman plots of ED_{50}, ED_{85}, ED_{mean} measured in the first and second investigator tests.

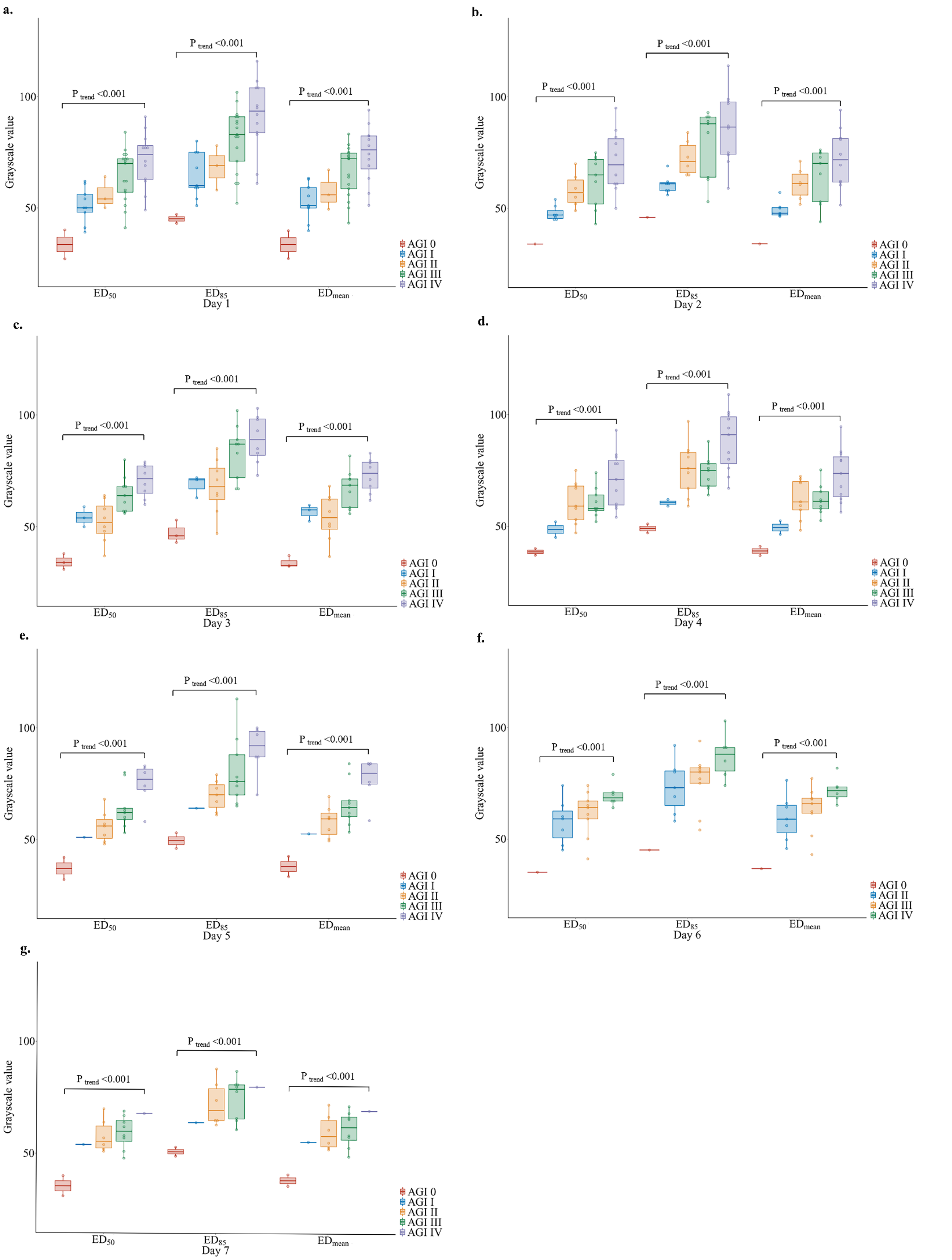
a.
b.
c.
d.
e.
f.
g.
Grayscale value
100
50
P trend <0.001
ED$_{50}$
ED$_{85}$
ED$_{mean}$
Day 1
Day 2
Day 3
Day 4
Day 5
Day 6
Day 7
AGI 0
AGI I
AGI II
AGI III
AGI IV

Figure S3. (a–g) Box plots of ED_{50}, ED_{85}, and ED_{mean} for varied AGI grades in the first 7 days after ICU admission. The trend test is mainly based on the median ED_{50}, ED_{85}, and ED_{mean} of each AGI grade on different days, which were input separately into the linear logistic regression model as continuous variables. AGI: acute gastrointestinal injury, ICU: Intensive care unit. *: *p*-value < 0.05.

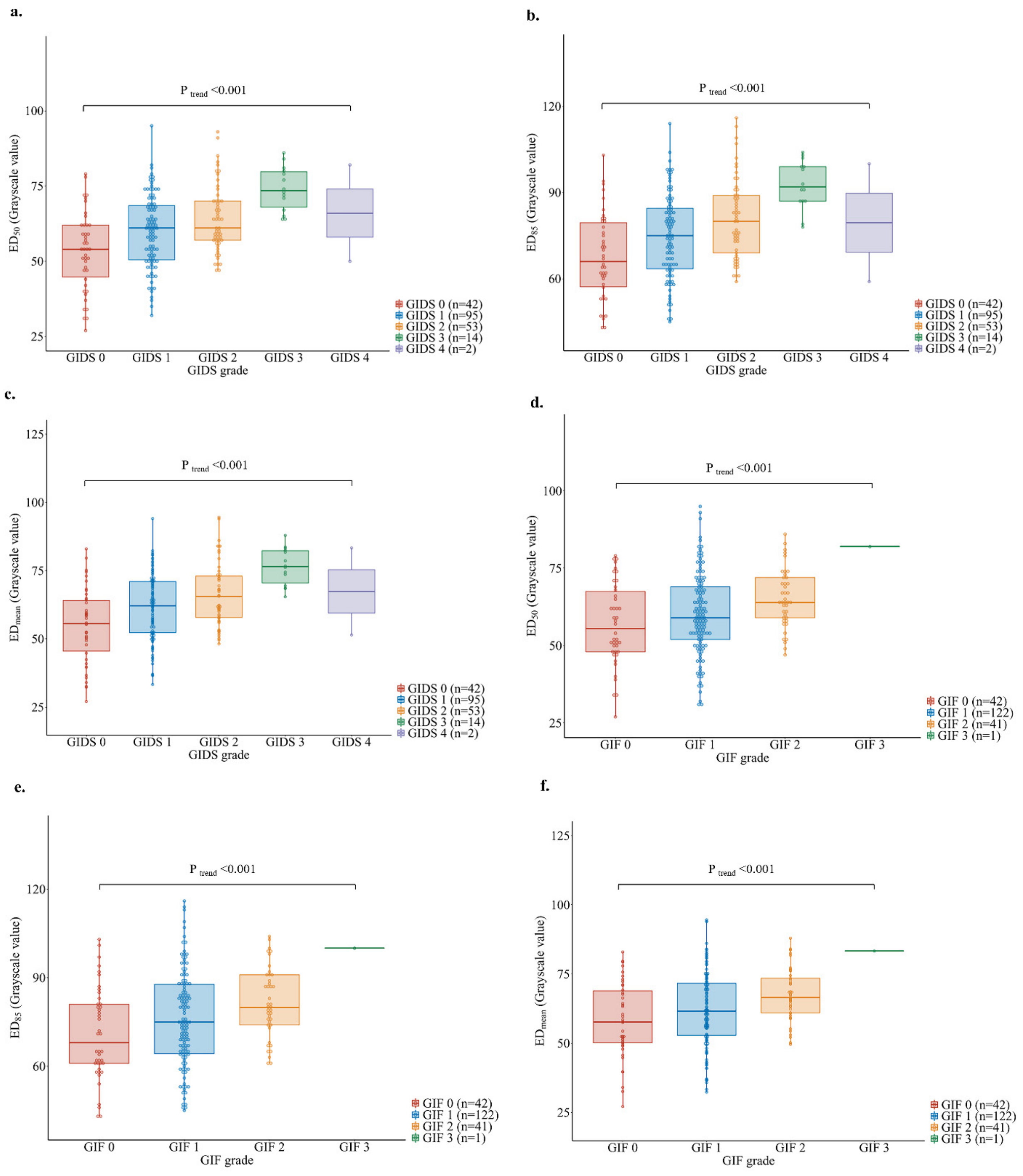

Figure S4. Boxplot of the change of ED_{50}, ED_{85}, ED_{mean} with GIDS and GIF score. The trend test is mainly based on the median ED_{50}, ED_{85}, and ED_{mean} of each GIDS grade **(a–c)** or GIF grade **(d–f)**, which were input separately into the linear logistic regression model as

continuous variables. GIDS score: Gastrointestinal Dysfunction Score; GIF score: Gastrointestinal Failure score.

Table S1. Detailed description of feeding intolerance. FI feeding intolerance

Variable	Total
FI occurrence—No. (%)	
Vomiting/regurgitation	10.0 (26.3%)
Abdominal distension	7.0 (18.4%)
Diarrhea	3.0 (7.9%)
Ileus	2.0 (5.3%)

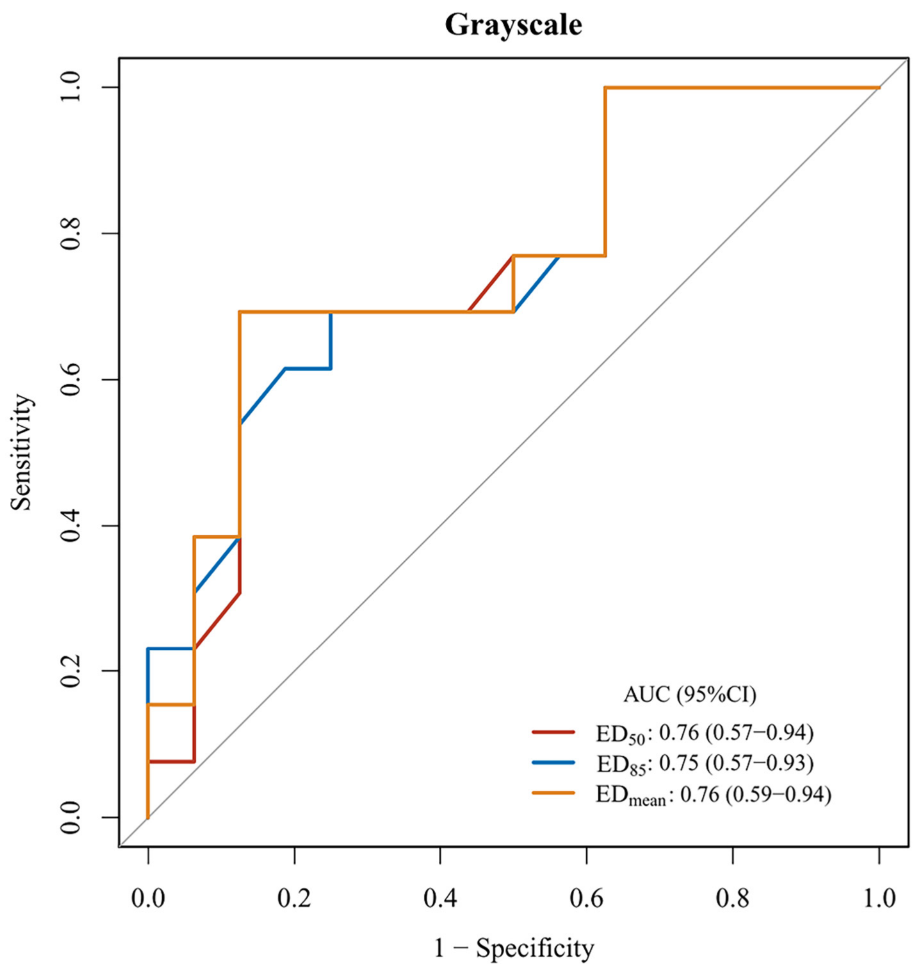

Figure S5. The receiver operating characteristic curve of ED_{50}, ED_{85} and ED_{mean} in predicting feeding intolerance at enteral nutrition initiation.
